# Older people’s perception of being frail – a qualitative exploration

**DOI:** 10.1186/s12877-024-05079-x

**Published:** 2024-05-23

**Authors:** Abigail J. Hall, Silviya Nikolova, Matthew Prescott, Victoria A. Goodwin

**Affiliations:** 1 Public Health and Sports Science Department, University of Exeter, Heavitree Road, Exeter, EX1 2LU UK; 2 Real World Methods & Evidence Generation, IQVIA, Reading, UK; 3 Bradford Teaching Hospitals NHS Foundation Trust, Bristol, UK

**Keywords:** Frailty, Older people, Qualitative, HERO

## Abstract

**Background:**

Frailty is a suggested consequence of ageing, but with a variety of different definitions the understanding of what it means to be frail is challenging. There is a common belief that frailty results in a reduction of physical functioning and ability and therefore is likely to significantly affect a person’s quality of life. The aim of this study was to explore the understanding of older people about the meaning of frailty and the potential consequences of being classified as frail.

**Methods:**

This paper forms a secondary analysis of a process evaluation of a complex intervention that was embedded within the individually randomised Home-based Extended Rehabilitation of Older people (HERO) trial. A maximum variation, purposive sampling strategy sought to recruit participants with a wide range of characteristics. Data collection included observations of the delivery of the intervention, documentary analysis and semi-structured interviews with participants. Thematic analysis was used to make sense of the observational and interview data, adopting both inductive and deductive approaches.

**Results:**

Ninety three HERO trial participants were sampled for the process evaluation with a total of 60 observational home visits and 35 interviews were undertaken. There was a wide range in perceptions about what it meant to be classified as frail with no clear understanding from our participants. However, there was a negative attitude towards frailty with it being considered something that needed to be avoided where possible. Frailty was seen as part of a negative decline that people struggled to associate with. There was discussion about frailty being temporary and that it could be reduced or avoided with sufficient physical exercise and activity.

**Conclusion:**

Our study provides insight into how older people perceive and understand the concept of frailty. Frailty is a concept that is difficult for patients to understand, with most associating the term with an extreme degree of physical and cognitive decline. Having a label of being “frail” was deemed to be negative and something to be avoided, suggesting the term needs to be used cautiously.

**Trial registration:**

ISRCTN 13927531. Registered on April 19, 2017.

## Background

Frailty is a clinical condition suggested to be an inevitable consequence of ageing [1] and despite no universally accepted definition, there is consensus that it involves susceptibility to external stressors such as physical, psychological and social factors [2], alongside a loss of biological reserve [3]. As such, even minor stressors can result in significant changes in health status [4]. A global ageing population, with an expected 2 billion of the world’s population predicted to be over 60 years of age by 2050 [5], will only exacerbate the challenges of conditions, such as frailty, with an increased prevalence in older populations. Therefore, understanding the challenges and consequences of frailty could be considered vital to improve healthy ageing and health and social care outcomes at an individual and societal level.

The multifaceted concept of frailty emphasises impacts on a person’s physical and psychological health and therefore the potential influence this has on social functioning – which are all factors reported to affect a person’s quality of life (QoL). Indeed, those living with frailty themselves have highlighted the importance in maintaining QoL rather than a focus on biomedical measures of outcomes relating to disease [6]. Therefore, the importance of understanding a person’s perception of their level of frailty and their resultant QoL is vital to understand how to target interventions to manage frailty alongside the medical management.

Existing literature focuses on older adults’ perceptions of frailty rather than the perceptions of those who are classified as being frail [7–11] as well as the difference between actually “being” frail and “feeling” frail [12]. There is a plethora of qualitative literature exploring perceptions of ageing in general, but with little focus specifically on frailty [13]. Similarly, there is literature exploring the perceptions of frailty from healthcare professionals [14, 15].

This paper forms a secondary analysis of a process evaluation (PE) of a complex intervention that was embedded within the Home-based Extended Rehabilitation of Older people (HERO) randomised controlled trial involving 742 participants living with frailty following a hospital admission for an acute illness or injury [16]. The process evaluation explored the community delivery of a complex intervention and involved a variety of different interacting components. This individualised, graded, and progressive 24-week exercise programme was delivered by National Health Service (NHS) physiotherapy teams to people aged 65 and older living with frailty. Frailty as an inclusion criteria for the HERO trial was identified using the Clinical Frailty Scale (CFS) [17] following discharge from hospital (+/- rehabilitation) after an acute illness or injury. The intervention incorporated behaviour change techniques based on social cognitive theory, including providing information on benefits of exercise; setting graded tasks; goal setting, problem solving, reorganising the physical environment to facilitate exercise, encouragement, feedback, prompting self-monitoring and rewards.

Evidence from the HERO trial suggests that there is incongruity between older people “being frail” and “feeling frail” [9, 12], and that feeling frail can have a large detrimental effect on the person’s wellbeing [18]. Thus, the person’s perception of their level of frailty has the potential to directly impact on their physical and psychological wellbeing. The aim of this paper was to explore the perceptions of frailty for those who were assessed to be frail. It will also consider how these perceptions of frailty affect an individual’s everyday functioning and QoL.

## Methods

The qualitative PE for the HERO trial employed a qualitative mixed methods approach [19] including a variety of data collection methods such as non-participant observations, semi-structured interviews, and documentary analysis. The data for this paper was obtained from the interviews with participants as the focus is on individual’s own perception of their frailty. Carers were often present at these interviews but contributed little to the data and the non-participant observations which allowed the researcher to observe the delivery of the intervention.

Social Cognitive Theory (SCT) [18] was used to explore the intervention and perceptions of frailty from the perspective of the participant and guided the development of the topic guides and subsequent analysis. SCT is extensively used in health and social care research and explains how individuals within social systems enact multiple human processes, including the acquisition and adoption of information and knowledge. Its focus was the interplay between personal factors, their behaviour, and their environments. The COREQ checklist [20] was used to report the study.

Research ethics committee approval was obtained from the Health Research Authority Yorkshire & The Humber – Bradford Leeds Research Ethics Committee – reference 17/YH/0097. All participants gave written informed consent to take part in the study.

### Recruitment

#### Inclusion criteria

HERO trial inclusion criteria are detailed in the trial protocol [16], but broadly included individuals who were:

- Age ≥ 65 years of age
- Community dwelling
- Acutely admitted to hospital with acute illness or injury then discharged home from hospital or associated rehabilitation
- Classified as mild, moderate, or severe frailty, defined as a score of 5–7 on the 9-item (CFS)
- Able to complete the Timed Up and Go test independently (i.e. stand from a chair and three meters before turning to return and sit in the chair)

HERO trial participants were asked to optionally consent to the trial PE. Those people consenting to PE activity were then purposively sampled to ensure all types of data collection included participants with a wide variety of experiences, demographics, and contexts to represent a wider perspective of the population under study. The researchers had no prior relationship with the participants. Participants were aware of the purpose of the research and the aims of the interviews. Participants were contacted via letter to request their involvement in the interviews. A maximum variation, purposive sampling strategy was used for the following characteristics:

- levels of frailty (Clinical Frailty Scale (CFS) levels 5–7)
- level of intervention (Home-based Older People’s Exercise (HOPE) programme levels 1–3)
- age
- sex
- intervention delivery site (NHS trust)

Recruitment to the process evaluation continued until it was felt no new themes were emerging and thus, we had sufficient information power [21] to answer our overall research questions and objectives.

HERO trial participants consenting to PE activity and randomised to receive the trial intervention were sampled and approached to participate in intervention delivery. These observations were scheduled to observe a variety of different participants at various stages of the intervention (from the first face to face home visit to the final face to face visit). While trial participants sampled for the PE had already consented to their involvement, permission to observe a therapy session was sought ahead of every observation from both the therapist and the participant.

Participant and carer interviews included trial participants in both the intervention and usual care arms of the trial. These interviews were scheduled approximately six months after an individual entered the HERO trial, and were timed to correspond with the intervention finishing for those randomised to receive it. Due to the lapse of time between consenting to participate in the HERO trial, and the scheduling of the process evaluation interviews, additional process evaluation participant information was provided and participants were asked to confirm consent prior to interview. Sampled participants were approached via a letter of invitation which was then followed up by a telephone call to discuss their potential involvement in a face-to-face interview. The interviews were all conducted in the participant’s own home and lasted for an average of 60 min. Two of the sampled participants refused an interview at this point – both reporting that they were unwell. Several of the participants we interviewed had carers or relatives present for the interview – the majority did not contribute to the interview and simply listened to the conversation.

#### Data collection

Topic guides were developed by the research team and used when undertaking interviews and used SCT to structure the lines of enquiry. All questions on the topic guide were used in every interview to ensure breadth of discussion. The two interviewers (AH and FZ) were both post-doctoral academics with extensive experience of qualitative research, including multiple publications and have both undertaken extensive training in qualitative research. The topic guides were developed to ensure that interviewees’ experiences were explored in relation to the underpinning theory relating to behaviour change. The topic guides were developed to explore many components of the intervention, but there were specific questions which related to frailty and the participants’ perception of what this meant to them. The topic guide and questions were initially piloted with several participants and after making small typographic changes, were then used for all other participants. Interviews were audio recorded, encrypted and later transcribed. Field notes were taken where appropriate. At the end of the interview, the participant was asked if they were happy for all their data to be included in the analysis. All data were pseudo-anonymised, and unique identification numbers associated with participants removed. All data were stored electronically on password protected secure servers. In order to ensure that each non-participation observation was explored consistently, a checklist and template was developed. This included observations of the delivery of the intervention, the environment as well as the interaction and relationship between the therapist(s) and the participant and carers.

#### Types of data collected

**Fig. 1:**
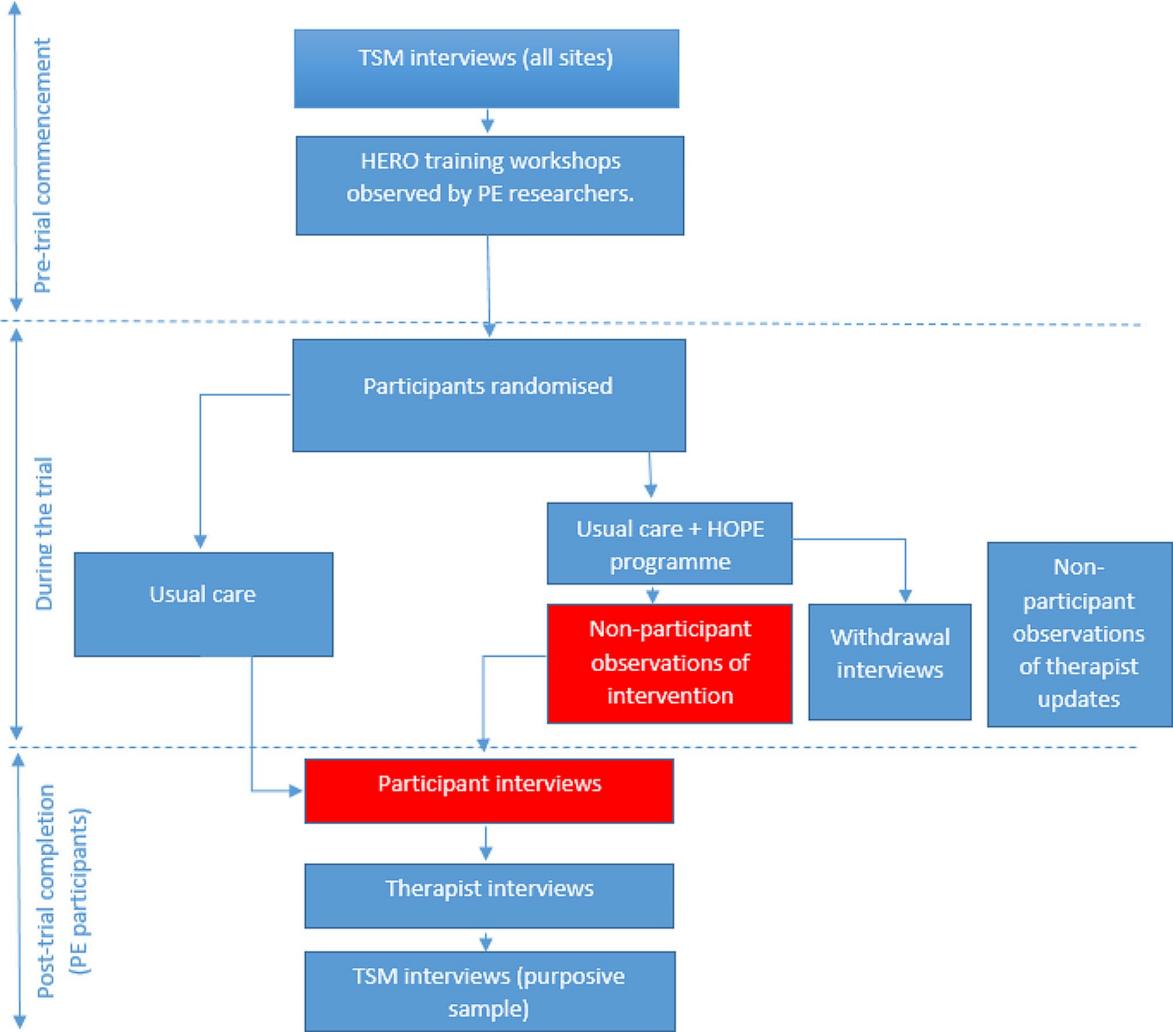
HERO trial Process evaluation activities (PE = process evaluation, TSM = therapy service manager, HOPE programme = The Home-based Older People’s Exercise programme)

Figure 1 depicts the data that were collected for the whole of the process evaluation, with the red boxes demonstrating the sources of data for this study. Data were collected as per the description in the trial protocol [16].

#### Analysis

An approach based on thematic analysis [22] was used to interpret the observational and interview data, adopting both inductive and deductive approaches. The first stage of data analysis involved the two researchers (AH and FZ) collecting the raw data from interview transcripts, field notes and patient observations. NVivo 11 (QSR International) was used to organise and store the data with all transcripts, observational frameworks and field notes uploaded to the software. Following completion of data collection, a first stage of coding was undertaken independently by each researcher. This resulted in preliminary codes for each set of participant data being generated and was reviewed jointly by the researchers and discussed with a third reviewer (DJC). This was part of a process of consensus building around the generation of themes. The themes that were being developed were continually reviewed in the context of the intervention logic model and used to develop a framework. Data were triangulated from all data sources to gain a clear understanding about any conflicting themes – for example, the observational data was compared to the data from the interviews and therapy record to determine if there was any conflicting data in what was observed and what was reported.

### Findings

In total, 93 HERO trial participants were sampled for the process evaluation, and their characteristics are detailed in Table 1. 38 intervention participants had their intervention delivery observed with a total of 60 observational home visits (the majority of participants had two observations) and 35 interviews were undertaken.

**Table 1:** Characteristics of participants sampled in the process evaluation

	Usual care *n* = 18	Intervention Participants *n* = 60	Withdrawal *n* = 15
**Age**			
Mean (± SD)	83.33 (6.29)	83.18 (7.77)	89.67 (3.74)
Median	83.00	83.00	90.00
Range	71–97	72–98	84–95
**Sex**			
n = Female	10	38	10
(%F)	55.56	63.33	66.67
**Frailty level**			
Mild	13	32	8
Moderate	5	26	7
Severe	0	2	0

One of the main criteria for inclusion in the HERO trial was an individual being classified as frail, however the main trial did not specifically inform people that they had been classified as ‘frail’ or discuss the CFS classification in such terms. As part of the PE data collection, we sought to determine general attitudes towards frailty as a term and concept, and determine whether those interviewed considered themselves as being frail. While some participants realised that they could be characterised as being frail and saw it as an inevitable consequence of ageing, others disputed it, feeling that it was a negative characteristic they didn’t wish to be associated with. There was also a feeling that frailty was a state that could fluctuate, it was a fluid state that could improve or worsen. Some participants felt they could control their level of frailty, others felt overwhelmed by it and daunted by the prospect of becoming frailer.

### Perception of frailty

Despite all our older main trial participants being classified as frail, there were very different attitudes towards frailty and perceptions of what being frail meant to them and to others. When asked to define what they understood “frail” to mean, the vast majority related frailty to a person’s physical abilities, thus if they felt they were physically able they would not perceive themselves to be frail.

Others related frailty to an image of what a frail individual might look like, often describing the image to be a thin, “fragile”, person who might look malnourished and be described to be “cognitively poor”. None of our participants described themselves as looking frail, indeed frailty wasn’t a concept that most found easy to identify with.

While participants often related frailty to physical difficulties, there was a belief expressed by a few of our participants that cognitive deficits (or lack of) contributed to their perception of frailty. Often, where an interviewee had some physically difficulties, if they perceived themselves to be cognitively sound, they didn’t feel they were frail – thus for some there is an element of needing both physical and cognitive deficits in determining whether somebody feels frail.

*Your abilities. Things that you can’t do that you used to do. I’m not frail in my mind….But when you know the things that, up to possibly a year ago, you could do and suddenly you’re unable to do them, and I had a dramatic weight loss. I mean, I’ve lost three stone. – (156, mild frailty)*

Pre-existing disabilities appeared to play a role in considering whether somebody was frail or not. Functional limitations relating to long standing disability were seen as separate when considering frailty, and thus did not predispose a person to being considered frail.

While some participants reported that their carers or family supported them to do functional tasks to aid building confidence, others were cautious about doing so and were fearful that enabling the person in this way may lead to over-confidence, which could pose a risk to their safety.

### Acceptance

There was a common belief that frailty related to a limitation in being able to do the tasks or activities that they wanted to be able to do. These tasks varied from simple activities of daily living that had become harder to more vigorous activities such as running. This inability to undertake a task provided very clear evidence that a person was physically deteriorating and often people related to a reduction in QoL and thus to an onset of frailty.

*You know when you want to run and you can’t run, and I think, I’m getting very frail now. (283 moderate frailty)*

*Just not being able to do things. Not being able to get on the bus and go to the supermarket, and being tired halfway around, and falling asleep all the time, I fall asleep through the day, I’ve… I’ll be watching something or doing something and my eyes just go, and I’m asleep for about an hour, which is a long time, and then when I go to bed I’m wide awake. – (18 mild frailty)*

Challenges undertaking functional tasks led people to seeking or accepting help. While some people felt that this inability to undertake functional activities classified them as being frail, others simply found the challenges associated with it frustrating, but didn’t feel this classified them as frail.

*Yes, well I mean I have needed help, I’ve got things that help in your kitchen and things like that, you know, to do different jobs that you can’t do anymore, you know, there’s certain things I can’t do, like even the things like the bleach bottles, you know where they have those locks on, I’m up a gum tree with them, I can’t press and do, my hands just aren’t doing it, so but I don’t think, I’ve never thought of that as frail, I just thought of that as a blooming nuisance, I have to get [son] to come, or somebody to come and do it you see (368 moderate frailty)*

Identifying as ’frail’ was problematic for many of our participants, however, there was evidence to suggest that their relatives and carers often told them that they were frail – frequently relating this to things that they were unable to do.

*P : He’s always saying “I can’t do this, I can’t do that”. (283 moderate frailty)*

Despite our participants all being classified as being “frail”, none of them overtly recognised themselves as being frail.

### Denial / avoidance

Our data indicated multiple negative connotations towards the term “frailty”. It was something that people perceived to be a negative characteristic and was something that they wanted to avoid, despite an acceptance that with increased age, came increased risk of frailty. The majority of participants we sampled in the process evaluation were classified as mild to moderately frail (CFS level 5 and 6), however, even the participants with severe frailty wished to avoid considering themselves as being frail.

*I know I must be getting frailer but I try not to think about it to be quite honest. I think, well if I can still do it I can’t be that frail can I. And that’s what I say to myself you see, I can’t be so bad if I can still do it so I’m going to try. – (154, severe frailty)*

There was an element of comparing themselves to other people who were less functionally able, and this appeared to give them comfort that they weren’t as frail as somebody else, or frail at all. If they knew people who were less physically able, this gave them confidence that they were doing well.

*I don’t think that frailty is, as such is an issue, and you know, see elderly people who are much less able than I am. – (321 mild frailty)*.

Participants related confidence to feelings of frailty. This confidence was important to enable them to undertake daily activities or things they enjoyed. Being unable to do these things was found to be associated with a reduction in QoL. Where they had lost confidence to undertake a task they previously could do, or participate in certain activities, they related this to a feeling of being frail. This loss of confidence was reported to have been as a result of injury or illness, or in some cases just as a result of general decline.

*I suppose I did, but then it comes back to confidence, if you’re not confident in doing something, whether that is a sign of frailty I don’t know, - (321 mild frailty)*

The participant often pointed out their cognitive abilities as a means to indicate they were not frail. The participant, in some cases, didn’t realise how many physical tasks they struggled to achieve until they were discussed and led them to reconsider whether they were indeed frail.

Many participants felt shame associated with classification of frailty and tried to mentally justify to themselves the reasons why they were not frail. It was perceived to be something to try and avoid as much as possible as it had significant negative connotations for a person’s QoL. The concept of QoL was raised by several participants and it was felt that frailty had a direct relationship with QoL – thus as a person got frailer, their QoL reduced.

*Well they wouldn’t be able to do anything at all would they, that’s how I feel, if you’re so frail. I mean, life’s not worth living if you get that frail is it. – (154 severe frailty)*

### Reversibility

While participants frequently struggled to accept being classified as frail, they could often relate to feelings of frailty at different stages of their life and how these were temporary. Several participants reflected on a specific period of ill-health or an injury and a perception of frailty, but as a transient problem. During this time they accepted that their functional ability and QoL would temporarily reduce, but this would only be short lived. Thus, the transient nature of frailty meant that they felt they had a level of control over it.

*I have been [frail] this last few months, because I’ve been in and out of the hospital, but normally I’m not a frail person, I’m not… (18 mild frailty)*

Other participants, despite being classified as frail, did not feel that such events should result in a classification of frailty and the temporal nature of deterioration didn’t relate to being frail and to be classified as frail required a long period of time.

There was a general belief that there were positive actions that could be taken to reduce frailty which most commonly was reported to be the use of exercise. Within the intervention participants, perceived improvements in physical ability relating to undertaking the HOPE programme led some people to believe that their level of frailty was being reduced.

*Um, well before I started doing the programme I felt really frail and then once I actually got into the programme itself I could feel meself getting stronger and stronger each time I were doing it and, but personally I really enjoyed doing it. – (235 mild frailty)*

Where people engaged with exercise, they noted improvements in functional ability which allowed them to do more activities that they enjoyed and thus resulted in an improvement in their QoL. Examples given included being able to engage more with their grandchildren or going out to cafes with friends and families. While exercise was seen to reduce frailty, there was a common theme that participants wanted to delay the onset or progression of frailty as much as possible. The negative connotations that people described related to ‘frail older people’ invoked fear about a reduction in their QoL and functional abilities and was something they wanted to avoid for themselves, thus there was a difficulty accepting a classification of frailty.

*Well I think when you can’t do what you used to do, I do think now, sometimes, I must admit sometimes I do feel a bit frail because I can’t do what I used to do … but it don’t go, but I do try. And I make meself go, I don’t sit and feel sorry for meself, I’ve never been that type, so I do try and make meself go as much as I can. – (33 mild frailty)*

## Discussion

The aim of this paper was to explore the perception of frailty from those who are classified as being frail. It also considered how individuals’ perception of frailty, may affect their everyday functioning and QoL. Our participants were all recruited to a large randomised controlled trial, with inclusion requiring classification as frail (score of 5–7 on the 9-item (CFS) [17]. Thus all our trial participants had at least a degree of functional dependence due to physical or cognitive deficits, yet their perceptions of what it meant to be frail varied and the effect this had on their attitudes to their QoL and physical abilities was also inconsistent. Most participants sampled in the process evaluation were either mild or moderately frail, however, those that were severely frail appeared to have greatest disconnect from considering themselves frail than those with lesser levels of frailty. While being frail reduces the ability to undertake functional tasks, participants reported getting help to undertake activities which allowed them to maintain their QoL.

One of the main findings of our study was a noted disconnect that people felt between being classified as frail and identifying as frail. The majority did not realise that they were classified as frail and reasoned why they should not be. Unanimously, our participants described frailty as being a negative state from both a physical and a psychological aspect, which is consistent with existing literature [7–9, 12, 23]. However, most participants did not have a clear idea what it meant to be frail, but still felt it was something they wanted to avoid.

In their study, Warmoth and colleagues [9] reported that participants felt that their frailty was “beyond their control”, however, many of our participants felt that frailty was actually transient in its nature and there were measures that could be undertaken to reduce the likelihood of becoming frail or to reverse it. These mainly revolved around undertaking exercise, or from ensuring that they continue to do activities – regardless of how difficult they found them. It is conceivable that our trial participants allocated to receive the HOPE programme had positive experiences of exercise impacting upon their feelings of control relating to frailty levels. Indeed, out trial population were rather self-selecting in that they had volunteered to participate in an RCT involving an exercise programme as extended rehabilitation. One might assume therefore that the participants valued rehabilitation and exercise as a component there of. Literature supports the notion of reversible frailty. A systematic review [24] of 46 studies with an included 15,690 participants suggests that frailty is reversible with a combination of muscle strength training and protein supplementation. A further systematic review and network meta- analysis [25] including 66 RCTs concluded that physical activity interventions, when compared to placebo and standard care, were associated with reductions in frailty.

Despite our participants reporting that physical frailty was potentially reversible, there was a belief that cognitive frailty was not. Cognitive frailty was believed to be a strong indicator as to classifying somebody as frail. Indeed, despite recognising their own physical difficulties, some of our participants relied on their cognitive abilities as a reason to not self-identify as frail. Wang and colleagues [26] explored the interdependency between cognitive frailty and physical frailty and suggested that early identification of cognitive frailty could facilitate specific interventions which could increase (or delay decline of) independence in older adults. The importance of maintaining independence was key to our participants. A person being classified as frail on the CFS, and them identifying as frail were often not consistent. The maintenance of physical abilities – and thus independence - appeared to reduce their feelings of being frail, participants had a tendency to focus on the preservation of abilities in reasoning why they we not frail, rather than recognising lost abilities. In instances where carers highlighted areas of dependence, some participants began to recognise a state of frailty.

Our participants were classified as being frail according to the CFS [17]. Although an interplay between physical frailty and dementia/cognitive decline is well recognised [27], the CFS focuses on function, an individual’s limitation of functions and dependence on others without differentiation between specific physical and cognitive deficits impacting the functional status. It is beyond the scope of this paper to compare measured cognitive and physical abilities of the HERO trial participants. Accepting that the status of individuals may have changed through the first 6 months involved in the trial pre-interview, all trial participants were sufficiently cognisant to provide informed consent to trial participation, and all scored ≥ 20 on the Montreal Cognitive Assessment as an eligibility criterion. Exercise has been suggested to be beneficial to improve both physical and cognitive frailty [28]. Furthermore, frailty is defined as a vulnerability to external stressors [3], however, there was no indication from our data that people perceived it to be about vulnerability, suggesting further disconnect between health care professionals’ classification of frailty and older people’s beliefs around a state of frailty.

## Implications

This study highlights several important factors for frailty research and engaging frail older people in healthcare services. Firstly, our data has highlighted how people find it hard to relate to the terminology around frailty, with many perceiving frailty to be an extreme near end of life state. However, in the UK, many services are termed “frailty” services, thus if people do not relate themselves to this term, it may result in a failure to engage with services that could be of benefit to them. Furthermore, our participants believed that frailty could be reversed or delayed with targeted interventions such as exercise. This has important implications for describing the benefits of exercise to this population.

## Conclusion

Frailty is a term used by healthcare professionals to describe a state of physical and mental vulnerability, however, there is a disconnect with how older people and health care professionals understand the term. Frailty as a concept used in healthcare, is difficult for older people to understand and identify with, with most frail older adults associating the term with an extreme degree of physical and cognitive decline. Having a label of being “frail” was something that was deemed to be negative and something to be avoided, suggesting the use of the term needs to be used cautiously. Some frail older people could recognise transient periods where they would identify themselves as frail, but felt able to control their level of frailty to some extent (particularly via exercise). A strong desire to avoid frailty was driven by negative attitudes towards their perceptions of frailty and the association with lower QoL.

## Data Availability

The datasets used and/or analysed during the current study are available from the corresponding author on reasonable request.

## Abbreviations

CFS: Clinical frailty scale
COREQ: Consolidated criteria for reporting qualitative research
HERO: Home-based Extended Rehabilitation for Older people
HOPE: Home-based Older People’s Exercise
GP: General Practitioner
NHS: National Health Service
NPT: Normalisation Process Theory
PE: Process evaluation
QoL: Quality of life
RCT: Randomised controlled trial
SCT: Social cognitive theory
TSM: Therapy service manager
